# GA-binding protein alpha ensures proper blastocyst development by suppressing SMAD3-mediated transforming growth factor-beta signaling

**DOI:** 10.1093/procel/pwaf083

**Published:** 2025-10-23

**Authors:** Junzhi Liao, Ying Ma, Yanjiang Liu, Rong Guo, Hongjie Yao

**Affiliations:** State Key Laboratory of Respiratory Disease, The First Affiliated Hospital of Guangzhou Medical University, Guangzhou Medical University, Guangzhou 510120, China; Joint School of Life Sciences, Guangzhou Medical University, Guangzhou 511436, China; Department of Basic Research, Guangzhou National Laboratory, Guangzhou 510005, China; Guangzhou Institutes of Biomedicine and Health, Chinese Academy of Sciences, Guangzhou 510530, China; Department of Basic Research, Guangzhou National Laboratory, Guangzhou 510005, China; State Key Laboratory of Respiratory Disease, The First Affiliated Hospital of Guangzhou Medical University, Guangzhou Medical University, Guangzhou 510120, China; Department of Basic Research, Guangzhou National Laboratory, Guangzhou 510005, China; State Key Laboratory of Respiratory Disease, The First Affiliated Hospital of Guangzhou Medical University, Guangzhou Medical University, Guangzhou 510120, China; Department of Basic Research, Guangzhou National Laboratory, Guangzhou 510005, China; State Key Laboratory of Respiratory Disease, The First Affiliated Hospital of Guangzhou Medical University, Guangzhou Medical University, Guangzhou 510120, China; Department of Basic Research, Guangzhou National Laboratory, Guangzhou 510005, China

**Dear Editor,**

The life of mammals begins with the fertilization of an egg by sperm, resulting in the formation of a zygote. This zygote then embarks on a complex developmental journey, undergoing successive rounds of cell division, differentiation, and growth, eventually giving rise to a fully formed organism (Rossant, 2018). This intricate process is tightly regulated by mechanisms such as cell polarity, epigenetic modifications, signaling pathways, metabolic cues, and key transcription factors (TFs) (Xu et al., 2021, 2025). The E26 transformation-specific factor (ETS) family is one of the largest known families of transcriptional regulators. Research into the ETS family has yielded significant insights into its functions and regulatory mechanisms in stemness maintenance, lineage specification, and embryonic development (Mirzadeh Azad et al., 2023; Yang et al., 2024; Zhang et al., 2023). The ETS-related transcription factor GA-binding protein alpha (GABPA), encoded by *Gabpa*, plays pivotal roles in various physiological processes, including cell cycle regulation, apoptosis, and differentiation (Ripperger et al., 2015; Yang et al., 2007). Deletion of *Gabpa* in mouse models leads to embryonic lethality during the pre-implantation stage (Ristevski et al., 2004). Furthermore, *Gabpa*-null mouse embryonic stem cells (mESCs) show significant suppression of proliferation and increased cell death (Ueda et al., 2017). However, the molecular mechanisms through which *Gabpa* regulates embryonic development remain poorly understood. In this study, we observed that *Gabpa* knockdown resulted in developmental abnormalities in blastocysts. *Gabpa* loss led to the downregulation of gene expression in the inner cell mass (ICM) and trophectoderm (TE) at E3.5, while resulting in the activation of the Transforming Growth Factor-beta (TGF-β) signaling pathway through the upregulation of *Smad3* during the 8-cell (8C) and morula stages. Suppressing the TGF-β signaling pathway’s aberrant upregulation alleviated the detrimental effects of *Gabpa* knockdown on embryonic development. These findings suggest that GABPA plays a crucial role in early mouse embryonic development by inhibiting the SMAD3-mediated TGF-β signaling pathway.

To explore the dynamic expression of GABPA during early embryonic development, we conducted a comprehensive analysis of published RNA-seq and Ribo-lite datasets from murine pre-implantation embryos (Wu et al., 2016; Xiong et al., 2022). Our analysis revealed a distinct temporal expression pattern of *Gabpa*, characterized by transcriptional upregulation following zygotic genome activation (ZGA), followed by a progressive decline after the 8C stage. This was further supported by Ribo-lite data showing the highest ribosome-protected fragment (RPF) of GABPA in the late 2-cell (L2C) stage and a dramatic decrease in subsequent developmental stages (Fig. S1A). Meanwhile, an assay for transposase-accessible chromatin using sequencing (ATAC-seq) indicates that the GABPA binding motif is significantly enriched at the 2C-8C and ICM stages of early mouse embryonic development (Wu et al., 2016). The observed spatiotemporal regulation of *Gabpa* strongly suggests its potential role as a key regulatory factor in orchestrating critical events during early embryonic development.

To delineate the functional role of *Gabpa* in early embryogenesis, we employed a loss-of-function approach by microinjecting gene-specific small interfering RNA (siRNA) into murine pronuclear (PN) stage 2/3 zygotes. Quantitative reverse transcription PCR (RT-qPCR) analysis revealed a significant reduction in *Gabpa* mRNA levels following siRNA treatment (Fig. 1A). Phenotypic analysis revealed that while the majority of *Gabpa*-depleted embryos successfully underwent compaction and developed into morphologically normal morulae, a subset exhibited developmental delay (Figs. 1B and S1C). Notably, 113 hours post-hCG administration (corresponding to the expanded blastocyst stage in control embryos), a proportion of *Gabpa*-deficient morulae either failed to progress to morphologically distinct blastocysts or displayed visible necrosis (Figs. 1B, 1C, S1B, and S1C). These collective findings demonstrate that GABPA serves as an essential regulator orchestrating developmental progression during early embryogenesis.

**Figure 1.**
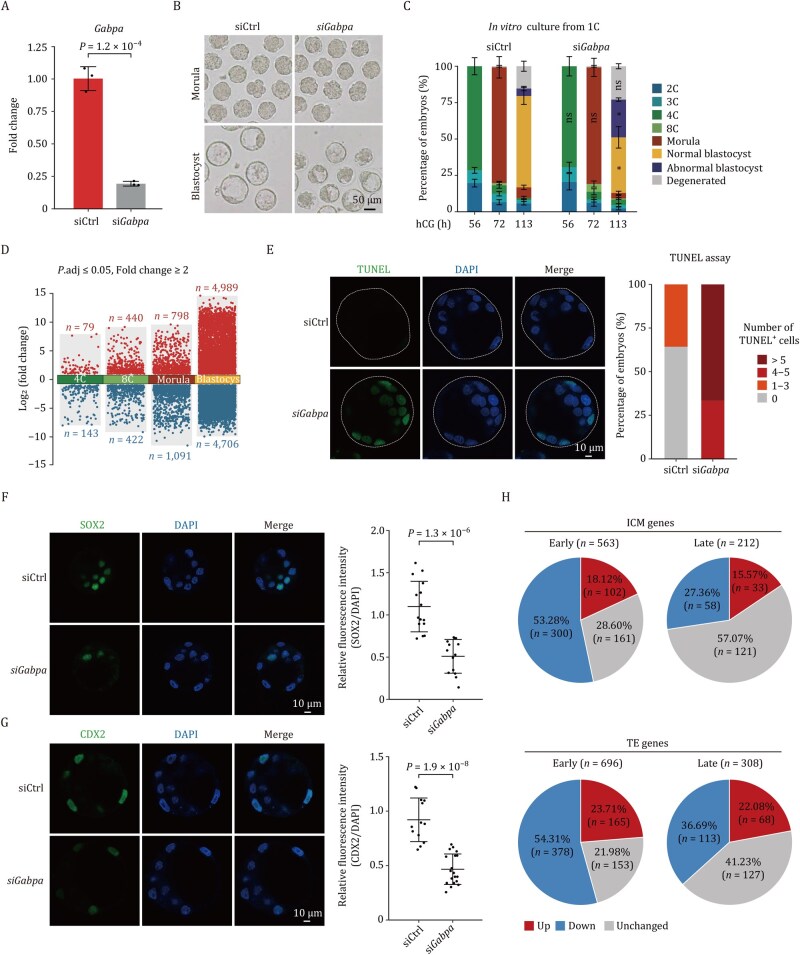
*Gabpa* regulates genes and early embryo development in mice. (A) RT-qPCR analysis of *Gabpa* expression in *Gabpa*-deficient embryos compared to control embryos at the E3.5 blastocyst stage. The data are represented as mean ± s.d. with significance determined by a two-sided *t*-test (*n *= 3 independent experiments). (B) Representative images of mouse embryos at the morula and E3.5 blastocyst stages of *Gabpa*-deficient and control embryos. Scale bar, 50 μm. (C) Developmental rates of *Gabpa*-deficient and control embryos during early *in vitro* development. The data are represented as mean ± s.e.m. with significance determined by a two-sided *t*-test (*n *= 3 independent experiments). (ns: not significant, *P < 0.05, **P < 0.01, ***P < 0.001). (D) Differential gene expression analysis of mouse pre-implantation embryos, highlighting upregulated (red) and downregulated (blue) genes. (E) Left, TUNEL assay and 4',6-diamidino-2-phenylindole (DAPI) for both siCtrl and *Gabpa*-deficient E3.5 blastocysts. Scale bar, 10 μm. Right, bar chart showing the percentages of TUNEL positive cells in siCtrl (*n *= 14) and *Gabpa*-deficient (*n *= 12) E3.5 blastocysts. The dashed square represents the entire blastocyst. (F) Left, immunofluorescence images of SOX2 and DAPI staining for siCtrl and *Gabpa*-deficient E3.5 blastocysts. Scale bar, 10 μm. Right, quantification of the relative fluorescence intensity of SOX2/DAPI in siCtrl (*n *= 15) and *Gabpa*-deficient (*n *= 14) E3.5 blastocysts. (G) Left, immunofluorescence images of CDX2 and DAPI staining for siCtrl and *Gabpa*-deficient E3.5 blastocysts. Scale bar, 10 μm. Right, quantification of the relative fluorescence intensity of CDX2/DAPI in siCtrl (*n *= 13) and *Gabpa*-deficient (*n *= 19) E3.5 blastocysts. The mean value is indicated by the horizontal line in (F) and (G). The error bar represents s.e.m. (H) The percentage of differential changes in early/late ICM and TE genes regulated in *Gabpa*-deficient E3.5 blastocysts. The early ICM/TE genes have an FPKM ≥ 1 in 8C embryos, while the late ICM/TE genes begin expression at E3.5 blastocysts and have an FPKM < 1 in 8C embryos.

To elucidate the molecular basis of *Gabpa’*s developmental regulation, we performed RNA sequencing (RNA-seq) on embryos treated with siRNA (Fig. S2A). Principal component analysis (PCA) of global transcriptional profiles demonstrated stage-specific clustering with intra-group consistency while maintaining clear inter-stage segregation (Fig. S2B). Intriguingly, *Gabpa* depletion caused a significant transcriptomic shift in E3.5, displacing them from their developmental trajectory in PCA space—an observation consistent with the morphological ­abnormalities observed (Figs. 1B and S2B). The most pronounced transcriptional changes in gene expression were observed at E3.5 (4,989 upregulated and 4,706 downregulated genes), partially reflecting the morphological defects (Fig. 1B–D). We then asked whether the transcriptional defects could be associated with developmental deficiencies. Gene ontology (GO) enrichment analysis revealed that the upregulated genes were primarily involved in key biological processes essential for embryonic development, such as nuclear division, chromosome segregation, and organelle localization (Fig. S2C). Conversely, the downregulated genes were primarily associated with previously characterized functions of *Gabpa*, including ribonucleoprotein complex biogenesis, non-coding RNA processing, and autophagy (Fig. S2C). After that, we noted that GO analysis revealed significant upregulation of biological processes associated with double-strand break repair and meiotic cell cycle, suggesting that the deficiency of *Gabpa* may promote apoptosis (Fig. S2C). Furthermore, a TUNEL assay demonstrated increased apoptosis in the *Gabpa*-depleted blastocysts (Fig. 1E). On the other hand, we performed 5-ethynyl-2'-deoxyuridine (EdU) incorporation assays to assess cell proliferation in *Gabpa*-deficient E3.5 blastocysts. The results showed that *Gabpa* knockdown did not significantly affect the proliferation of embryonic cells (Fig. S2D). To investigate the potential impact of *Gabpa* knockdown on the ICM and TE, we evaluated the expression levels of SOX2 (ICM marker) and CDX2 (TE marker), respectively. In line with the morphological defects, a considerable decline in both SOX2 and CDX2 protein levels was observed in the *Gabpa*-depleted blastocysts (Fig. 1F and 1G). Further analysis revealed that both ICM genes (53.28% in the early ICM and 27.36% in the late ICM) and TE genes (54.31% in the early TE and 36.69% in the late TE) were downregulated in *Gabpa*-depleted blastocysts, indicating that GABPA plays an essential role in blastocyst establishment (Figs. 1H, S2E, and Table S1). Taken together, these findings underscore the crucial role of GABPA in both establishing and maintaining blastocyst embryogenesis.

The developmental defects observed at the E3.5 blastocyst stage may result from cumulative transcriptional dysregulation initiated at earlier embryonic stages. Transcriptome perturbations were also observed at earlier stages, including the 4-cell (4C), 8C, and morula stages, even though the mutant embryos appeared morphologically normal (Figs. 1C and S1C), which might reflect the cumulative effects of *Gabpa* knockdown. To investigate this hypothesis, we conducted a temporal analysis of stage-specific differentially expressed genes (DEGs) following *Gabpa* knockdown during early embryogenesis. Our analysis identified 87 upregulated and 144 downregulated genes that were consistently shared between the 8C and morula stages (Fig. 2A), highlighting their crucial roles in *Gabpa*-mediated developmental regulation. GO analysis revealed significant enrichment of 87 consistently upregulated genes in biological processes related to the regulation of TGF-β signaling, suggesting a functional link between *Gabpa* and this critical signaling pathway (Fig. 2B). The TGF-β signaling pathway plays a pivotal role in early embryogenesis, particularly in maintaining pluripotency and guiding lineage specification in embryonic stem cells (Beyer et al., 2013). This signaling cascade is mediated through distinct SMAD proteins: TGF-β signaling primarily activates SMAD2/3, while BMP signaling functions through SMAD1/5/8. Intriguingly, transcriptomic profiling revealed sustained upregulation of *Smad3* (fold change ≥2) in *Gabpa*-deficient embryos at both the 8C and morula stages compared to control embryos (Fig. 2C). This transcriptional elevation was corroborated through RT-qPCR validation at the 8C stage, which mirrored the RNA-seq findings (Fig. 2C and 2D). The temporal dysregulation of *Smad3* during these pivotal developmental transitions implies that aberrant *Smad3* overexpression, resulting from *Gabpa* depletion, may constitute a key molecular mechanism underlying the observed embryonic developmental arrest.

**Figure 2.**
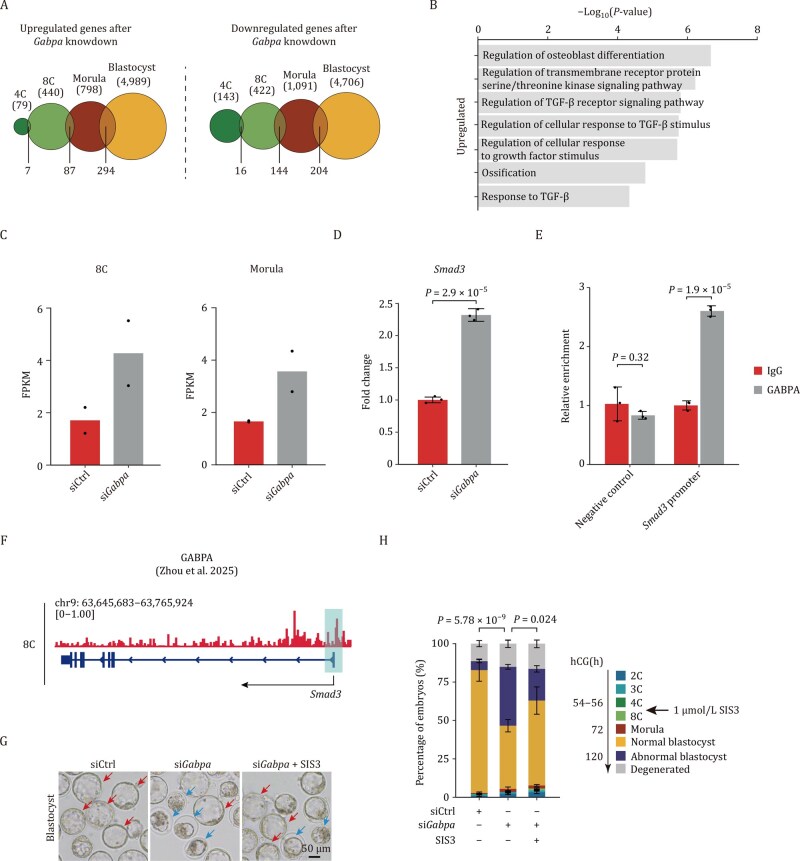
*Gabpa*-deficiency arrests embryo development via the upregulation of TG F-β/SMAD pathway. (A) Overlap of DEGs across different developmental stages. (B) GO analysis of the upregulated DEGs in the overlap between the 8C and morula stages in *Gabpa*-­deficient and control embryos. (C) RNA expression level (FPKM) of *Smad3* at the 8C and morula stages in *Gabpa*-deficient and control embryos (two biological replicates). (D) RT-qPCR analysis of *Smad3* in *Gabpa*-deficient embryos compared to control embryos at the 8C stage. (E) CUT&RUN-qPCR analysis showing the enrichment of GABPA at the promoter region of *Smad3* in 8C stage embryos. (F) Genome browser view showing GABPA binding to the promoter region of *Smad3* at the 8C stage. (G) Representative images of *in vitro* SIS3 treatment at the E3.5 blastocysts. Red arrowheads indicate normally developmental blastocysts, while blue arrowheads indicate abnormally developmental blastocysts. Scale bar, 50 μm. (H) Developmental rates following *in vitro* SIS3 treatment at the E3.5 blastocyst. The data are represented as mean ± s.e.m. with significance determined by the chi-squared test (*n *= 3 independent experiments). For (D) and (E), data are represented as mean ± s.d. with significance determined by a two-sided *t*-test (*n *= 3 independent experiments).

To investigate whether GABPA directly binds to and regulates *Smad3*, we employed CUT&RUN-qPCR to detect the enrichment of GABPA at the *Smad3* promoter region at the 8C stage. The CUT&RUN-qPCR results showed significant enrichment of GABPA at the *Smad3* promoter region (Fig. 2E). This finding is consistent with recently published data showing that GABPA directly binds to the *Smad3* promoter region during this stage (Zhou et al., 2025) (Fig. 2F). This result suggests that SMAD3 serves as a crucial downstream factor through which GABPA regulates embryonic development. To determine whether GABPA deficiency leads to *Smad3* overexpression and contributes to embryonic developmental defects, we employed the selective SMAD3 inhibitor SIS3 to block TGF-β-mediated SMAD3 phosphorylation (Jinnin et al., 2006). Given the observed *Smad3* upregulation starting at the 8C stage, we administered 1 μmol/L SIS3 to *Gabpa*-depleted embryos from the 4C stage, while control embryos were treated with DMSO. Pharmacological inhibition of SMAD3 phosphorylation significantly reduced the incidence of abnormal blastocyst formation, from 38.31% to 20.64% (Figs. 2G, 2H, S3A, and S3B). These findings establish a functional link between GABPA*-*mediated regulation of TGF-β/SMAD3 signaling and early developmental progression in mouse embryos.

The TGF-β signaling pathway serves as a critical regulator of organismal homeostasis, with its dysregulation implicated in diverse pathological conditions. Despite its recognized importance, the regulatory mechanism of the TGF-β signaling pathway in early embryos remains poorly understood. In this study, we provide functional evidence that GABPA suppression of SMAD3-mediated TGF-β signaling is essential for pre-implantation embryo development. Deletion of *Gabpa* leads to dysregulation of the transcriptome, embryonic developmental arrest, and defects. At the 8C and morula stages, we identified 87 genes that were consistently upregulated following *Gabpa* depletion. GO analysis revealed that these genes are associated with the TGF-β signaling pathway. Furthermore, treatment with the TGF-β inhibitor SIS3 significantly reduced the incidence of abnormal blastocyst formation caused by *Gabpa* knockdown. Collectively, this study positions GABPA as a key upstream regulator that orchestrates developmental progression by precisely modulating the TGF-β pathway.

## Supplementary data

Supplementary data is available at *Protein & Cell* online https://doi.org/10.1093/procel/pwae015.

## Footnotes

This work was supported by the National Key R&D Program of China (2021YFA1100300), and the National Natural Science Foundation of China (32430016, U21A20195, 32500489, 32570685).

The authors declare no conflicts of interest. All the authors agreed to participate in this paper and publish this manuscript.

All animal studies and experimental procedures were approved by the Committee on the Ethics of Animal Experiments of Guangzhou Institutes of Biomedicine and Health, Chinese Academy of Sciences. Informed consents were obtained from all the authors.

RNA-seq data are deposited in GSA (CRA023562). Public data used in this study: RNA-seq data of mouse MII oocyte to ICM: GSE66390. Ribo-lite data of mouse MII oocyte to ICM: GSE165782. GABPA binding in mouse embryos: GSE263171. All data needed to evaluate the conclusions are presented in the paper. Additional data is available from the authors upon request.

All authors reviewed and approved the paper. H.Y. conceived the study. J.L. and Y.M. collected samples and performed drug treatments. J.L. performed RNA-seq, qPCR, immunostaining, TUNEL assay, EdU incorporation and CUT&RUN-qPCR, analyzed the data. Y.L. and R.G. provided technical assistances. H.Y. and J.L. wrote the manuscript.

## Supplementary Material

pwaf083_Supplementary_Data
