# Supplementary material for: GA-binding protein alpha ensures proper blastocyst development by suppressing SMAD3-mediated transforming growth factor-beta signaling
Source: Protein Cell. 2025 Oct 23;17(3):263–7. doi: 10.1093/procel/pwaf083 (PMC12987565; doi:10.1093/procel/pwaf083)
Supplement: pwaf083_Supplementary_Data [file pwaf083_supplementary_data.zip › Suppementary materials.docx]

**I: Materials and Methods**

**II: Supplementary Figures and Figure legends**

**I: Materials and Methods**

**Animal maintenance**

Wild-type C57BL/6J mice were obtained from GemPharmatech Co., Ltd. (Guangdong, China), while DBA/2N mice were sourced from Vital River (Beijing, China). All animals were housed under specific pathogen-free (SPF) conditions with controlled environmental parameters: a 12-h light/dark cycle, ambient temperature maintained at 20-22°C, and ad libitum access to food and water. Animal care and experimental protocols were approved by the Institutional Animal Care and Use Committee (IACUC) of the Guangzhou Institutes of Biomedicine and Health, Chinese Academy of Sciences (Approval No. 2024050).

**Early embryo collection**

To obtain preimplantation embryos from mice, 4-8-week-old C57BL/6J female mice were intraperitoneally injected with pregnant mare’s serum gonadotropin (PMSG; 10 IU) and human chorionic gonadotropin (hCG; 10 IU) at 46-48 h after the PMSG injection. Following mating with DBA/2N males, preimplantation embryos were collected in M2 medium at the following time points after hCG injection: 21 h (PN2/3), 46 h (late 2C), 56 h (4C), 68-70 h (8C), 72 h (morula) and 92-94 h (blastocyst).

**Microinjection**

*Gabpa* siRNA sequences were meticulously designed to target distinct regions within mouse *Gabpa* mRNA. Microinjection procedures were conducted in the M2 medium. An injection pipette containing a 20 mM siRNA solution was inserted into the cytoplasm of the zygotes. Approximately 40 zygotes were microinjected and cultured to the blastocyst stage in KSOM medium at 37°C under 5% CO_2_ in air for subsequent collection or recording. The negative control siRNA oligo (sense sequence: 5’-UUCUCCGAACGUGUCACGUTT-3’; antisense sequence: 5’-ACGUGACACGUUCGGAGAATT-3’), *Gabpa*-siRNA-1 (sense sequence: 5’-GCUCAAGUGAUAACUCUUGTT-3’; antisense sequence: 5’-CAAGAGUUAUCACUUGAGCTT-3’), *Gabpa*-siRNA-2 (sense sequence: 5’-GCCAGGCCAUAGACAUCAATT-3’; antisense sequence: 5’-UUGAUGUCUAUGGCCUGGCTT-3’), *Gabpa*-siRNA-3 (sense sequence: 5’-AGAAGACAGAAGUUCACCGTT-3’; antisense sequence: 5’-CGGUGAACUUCUGUCUUCUTT-3’).

**Quantitative PCR with reverse transcription (RT-qPCR)**

Pools of 10 zygotes or embryos at various developmental stages were collected. Total RNA was extracted, and reverse transcription was performed using the EZ-press Single Cell to cDNA Kit (EZB, B0011) in accordance with the manufacturer’s instructions. Quantitative PCR was conducted using 2 × RealStar Fast SYBR qPCR Mix (GenStar, A301-10) on a CFX96 Touch Real-Time PCR Detection System (Bio-Rad) by following the manufacturer’s guidelines. The data were analyzed using the ΔΔCt method, with *Gapdh* serving as the internal control. All experiments were repeated three times. The primers used for the RT-qPCR assays are listed in Supplementary Table S2.

**SIS3 treatment**

The SMAD3 phosphorylation inhibitor (Selleck, S7959) was dissolved in DMSO to prepare a 10 mM stock solution. For embryo treatment starting at the 4-cell stage, SIS3 was diluted in KSOM to a final concentration of 1 μM. Embryos were washed at least three times with KSOM containing SIS3 before being cultured in the same medium for further development.

**RNA-seq library preparation and sequencing**

The RNA-seq libraries were generated from embryos using Geo-seq as described previously with minor modification (Chen et al., 2017). The zona pellucida was gently removed by treatment with Tyrode’s solution (Sigma, T1788). 10-20 embryos per sample were washed three times in M2 medium and then lysed in GuSCN (guanidine isothiocyanate) solution, with polyadenylated mRNAs captured using PolyT primers. The RNA samples were subsequently incubated for denaturation at 72°C for 3 min before reverse transcription. Following pre-amplification and purification with AMPure XP beads, libraries were generated using the TruePrep DNA Library Prep Kit V2 for Illumina (Vazyme, TD503) in accordance with the manufacturer’s instructions. All libraries were sequenced on the Illumina HiSeq 2000 following the manufacturer’s guidelines.

**RNA-seq data processing**

The raw sequencing reads were trimmed using Trim Galore (version 0.6.10) (Martin, 2011) to remove sequencing adapters. Subsequently, the reads were mapped to the mm10 genome using STAR (version 2.7.0) (Dobin et al., 2013), which has been shown to be highly effective for mapping. Gene expression levels were quantified with RSEM (version 1.3.1) (Li and Dewey, 2011). To identify differentially expressed genes, the DESeq2 package (version 1.40.2) in R was used (Love et al., 2014). Differentially expressed genes were identified using an adjusted P-value cutoff of 0.05 and a fold change cutoff of 2. GO enrichment analysis was performed using the R package clusterProfiler (version 4.8.3) (Wu et al., 2021).

**TUNEL assay**

Mouse embryos were fixed with 4% paraformaldehyde (PFA, Sigma-Aldrich, P6148) for 30 min, followed by three washes with PBS/0.1% Triton X-100/0.2% BSA. Next, the embryos were permeabilized with PBS/0.5% Triton X-100/0.2% BSA for 1 h. The TUNEL assay was performed using the One Step TUNEL Apoptosis Assay Kit (Beyotime, C1086) according to the manufacturer’s instructions. The confocal microscope (Zeiss, LSM900) was used for the detection of fluorescence. The numbers of TUNEL positive cells were quantified using hand counting after image capture.

**EdU incorporation**

Embryos were obtained and cultured in KSOM containing 10 μM EdU at 37°C for 2 h before fixation. Incorporated EdU was detected using BeyoClick™ EdU Cell Proliferation Kit with AF594 (Beyotime, C0078) according to the manufacturer’s protocol.

**Immunostaining and** **confocal microscope**

Mouse embryos were fixed with 4% PFA for 30 min, followed by three times of washing with PBS/0.1% Triton X-100/0.2% BSA. The samples were then permeabilized with PBS/0.5% Triton X-100/0.2% BSA for 1 h. After permeabilization, the embryos were blocked with PBS/3% BSA for 1 h. Subsequently, the embryos were incubated at 4°C overnight with primary antibodies (CDX2, at a ratio of 1:400, Abcam, ab76541; SOX2, at a ratio of 1:50, CST, 23064S). After three washes with PBS/0.1% Triton X-100/0.2% BSA, the embryos were incubated with secondary antibody Goat Anti-Rabbit IgG H&L (Alexa Fluor 488) at a ratio of 1:500 (Abcam, ab150077) at room temperature (RT) for 1 h. DNA was stained with DAPI (Abcam, ab104139) at RT for 1 h. All immunofluorescence images were taken by confocal microscope (Zeiss, LSM900) and were analyzed using ImageJ.

**CUT&RUN followed by qPCR**

The cleavage under targets and release using nuclease (CUT&RUN) -qPCR was performed according to the Hyperactive pA/G-MNase CUT&RUN Assay Kit for PCR/qPCR (Vazyme, HD103) with minor modifications. Briefly, the zona-free embryos were resuspended in 50 μl washing buffer with the activated Concanavalin A magnetic beads at RT for 10 min, then samples were fixed with 1% formaldehyde for 1 min. After performing three washes with washing buffer, the samples were incubated at 4°C overnight with anti-GABPA antibody (Proteintech, 21542-1-AP) or anti-IgG antibody (Sigma-Aldrich, 12-370). Samples were incubated with pA/G-MNase at 4°C for 1 h. Subsequently, samples were incubated with pre-cooled CaCl_2_ on ice for 1.5 h. Then, the stop solution was added to the reaction and incubated at 37°C for 30 min. Then, 2.5 μl 10% SDS and 2.5 μl 20 mg/ml Proteinase K (Tiangen, RT403) were added and incubated at 55°C for at least 2 h for reverse crosslinking. The DNA was purified by using the DNA Extract Beads (Vazyme, N411). Subsequently, the extracted DNA underwent RT-qPCR analysis, with spike-in DNA serving as the internal control. The primers used for the CUT&RUN-qPCR assays are listed in Supplementary Table S3.

**References:**

Chen J, Suo S, Tam PPL*, et al.* Spatial transcriptomic analysis of cryosectioned tissue samples with Geo-seq. *Nat Protoc* 2017;**12**:566-580.

Dobin A, Davis CA, Schlesinger F*, et al.* STAR: ultrafast universal RNA-seq aligner. *Bioinformatics* 2013;**29**:15-21.

Li B, and Dewey CN. RSEM: accurate transcript quantification from RNA-Seq data with or without a reference genome. *BMC Bioinformatics* 2011;**12**:323.

Love MI, Huber W, and Anders S. Moderated estimation of fold change and dispersion for RNA-seq data with DESeq2. *Genome Biol* 2014;**15**:550.

Martin MJEj. Cutadapt removes adapter sequences from high-throughput sequencing reads. *EMBnetjournal* 2011;**17**:3.

Wu T, Hu E, Xu S*, et al.* clusterProfiler 4.0: A universal enrichment tool for interpreting omics data. *The Innovation* 2021;**2**:100141.

**II: Supplementary Figures and Figure legends**

**
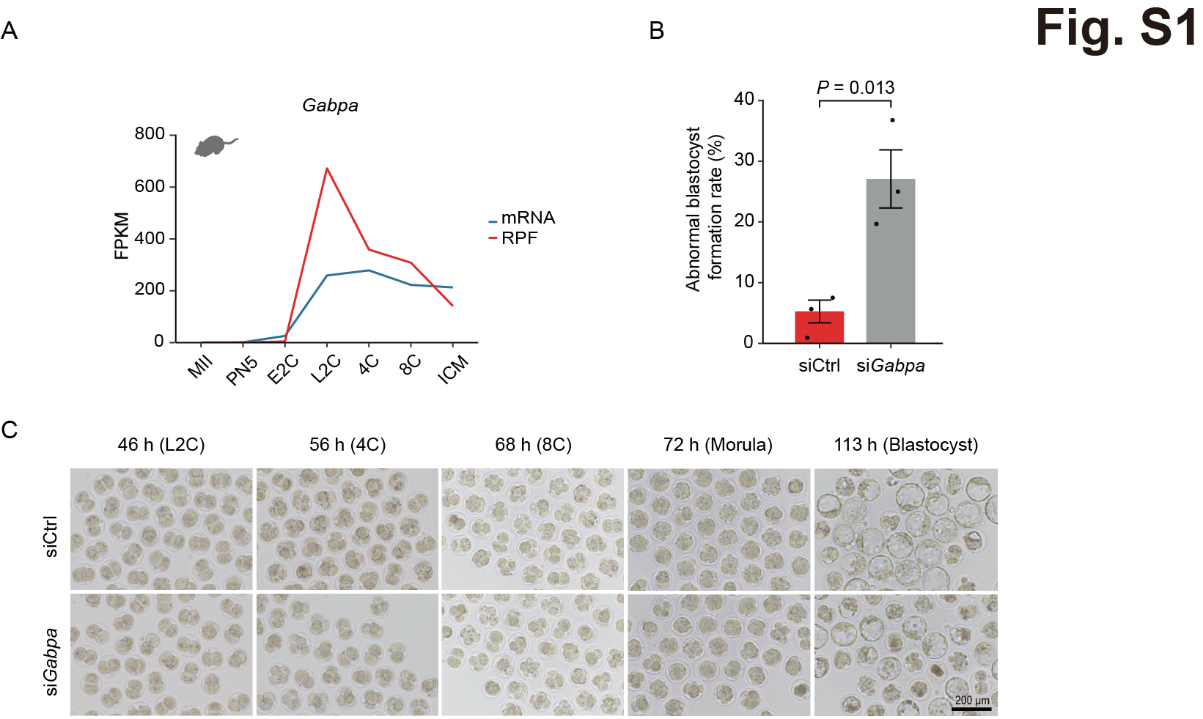
**

**Figure S1. *Gabpa*-deficiency causes abnormal early embryo development in mice.** (A) *Gabpa* mRNA levels from RNA-seq and RPF levels from Ribo-seq in both mouse oocytes and early embryos. (B) The abnormal developmental rate of blastocysts in *Gabpa*-deficient embryos compared to control embryos cultured *in vitro*. The data are represented as mean ± s.e.m. with significance determined by a two-sided *t*-test (n = 3 independent experiments). (C) Embryo morphology of control and *Gabpa*-deficient embryos at 46 h, 56 h, 68 h, 72 h, and 113 h, respectively, after hCG injection. Scale bar, 200 μm.


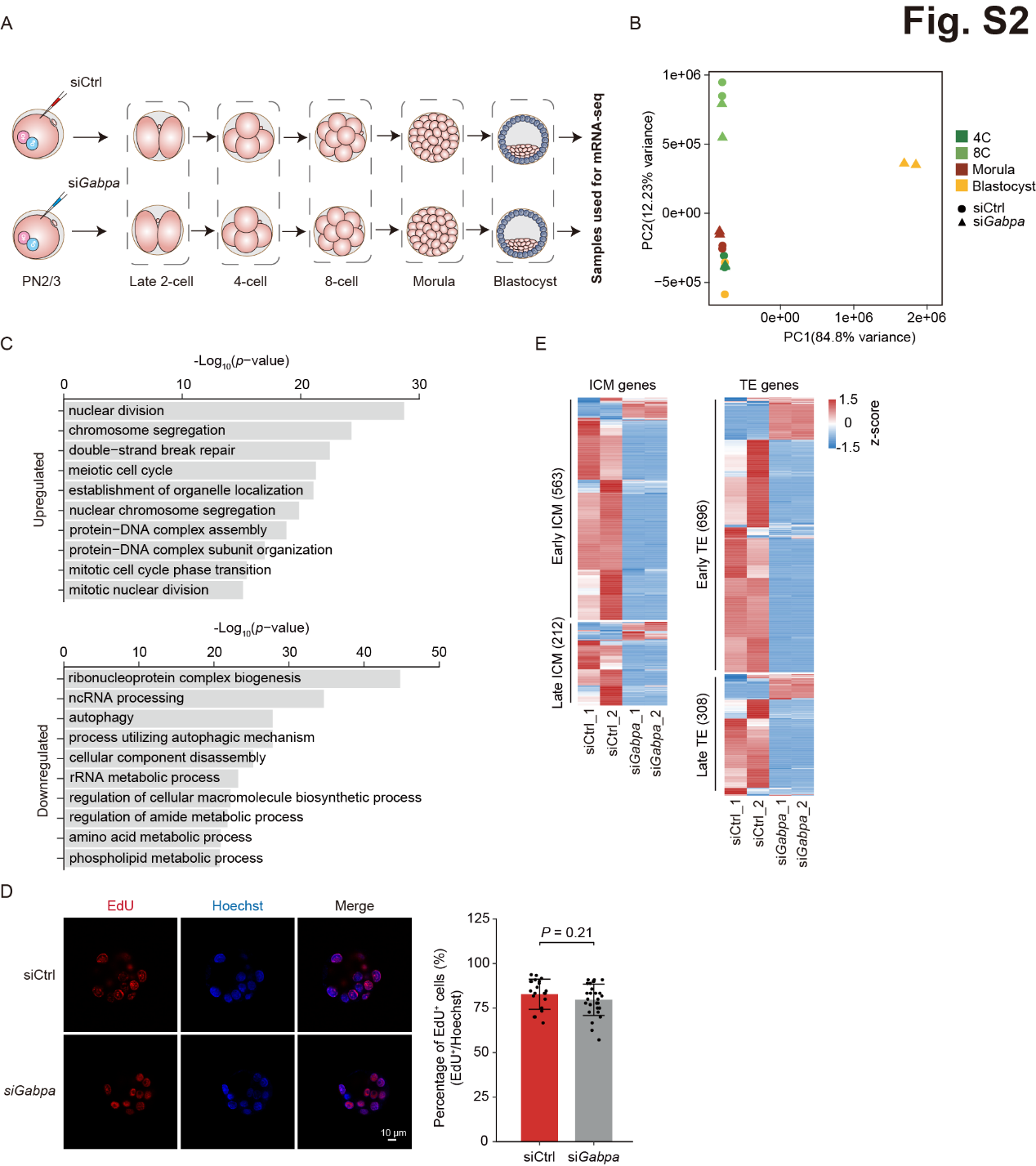


**Figure S2.** ***Gabpa*-deficiency alters gene expression.** (A) Schematic diagram illustrating the procedure for sample collection in embryos subjected to *Gabpa* knockdown. (B) PCA assessing of gene expression patterns from RNA-seq data during pre-implantation development under control and *Gabpa*-depleted conditions. (C) GO analysis of DEGs at the E3.5 blastocyst stage in *Gabpa*-deficient embryos compared to control embryos. (D) Left, representative images of EdU staining and Hoechst in siCtrl and *Gabpa*-deficient E3.5 blastocysts. Right, bar charts showing the percentages of EdU positive cells in siCtl (n = 22) and *Gabpa*-deficient (n = 28) E3.5 blastocysts. The error bars represent s.e.m. Scale bar, 10 μm. (E) Heatmap illustrating changes in gene expression in both ICM and TE in E3.5 blastocysts following *Gabpa* knockdown, compared to the control embryos.


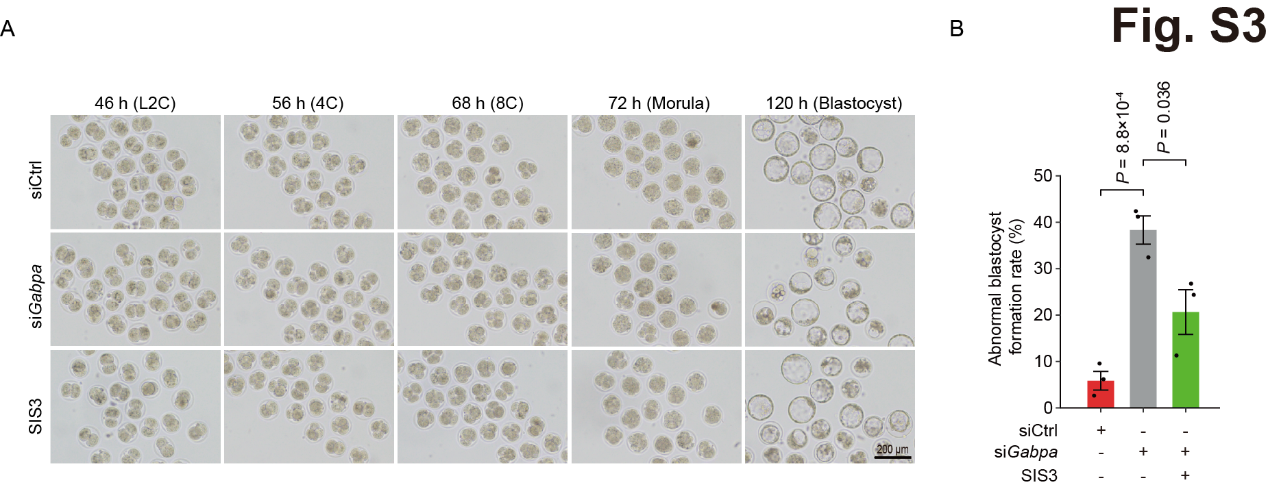


**Figure S3. SIS3 treatment rescues the impact of *Gabpa*-deficiency on embryo development.** (A) Representative images showing embryo morphology following treatment with siCtrl, si*Gabpa*, and si*Gabpa* combined with SIS3 at the indicated time points after hCG injection. Scale bar, 200 μm. (B) Quantification of abnormal blastocyst development rates following *in vitro* SIS3 treatment. The data are represented as the mean ± s.e.m. with significance determined by a two-sided *t*-test (n = 3 independent experiments).
